# Knowledge, attitudes, and practices regarding nutrition among patients with malignant tumors

**DOI:** 10.3389/fnut.2026.1741346

**Published:** 2026-01-21

**Authors:** Yuejun Ren, Jie Li, Jingjing Wu, Qiaohong Niu, Xiaolu Ren, Qiaoli Ren

**Affiliations:** 1 Medical Imaging Department, Shanxi Province Cancer Hospital/Shanxi Hospital Affiliated to Cancer Hospital, Chinese Academy of Medical Sciences, Cancer Hospital Affiliated to Shanxi Medical University, Taiyuan, Shanxi, China; 2 Department of Radiation Oncology, Shanxi Province Cancer Hospital/Shanxi Hospital Affiliated to Cancer Hospital, Chinese Academy of Medical Sciences, Cancer Hospital Affiliated to Shanxi Medical University, Taiyuan, Shanxi, China; 3 Department of Nursing, Shanxi Province Cancer Hospital/Shanxi Hospital Affiliated to Cancer Hospital, Chinese Academy of Medical Sciences, Cancer Hospital Affiliated to Shanxi Medical University, Taiyuan, Shanxi, China; 4 Department of Respiratory Medicine, Shanxi Province Cancer Hospital/Shanxi Hospital Affiliated to Cancer Hospital, Chinese Academy of Medical Sciences, Cancer Hospital Affiliated to Shanxi Medical University, Taiyuan, Shanxi, China

**Keywords:** knowledge, attitudes, and practices, nutrition, malignant tumors, patient, cross-sectional study

## Abstract

**Objectives:**

This study aimed to investigate the knowledge, attitudes, and practices (KAP) regarding nutrition among patients diagnosed with malignant tumors.

**Methods:**

A cross-sectional survey was conducted from 16 October 2023 to 30 April 2024, in author’s hospital. Demographic information and KAP were collected through structured questionnaires.

**Results:**

The study analyzed 535 patients. Males were 370 (69.16%), and 213 (39.81%) reported nutritional supplement use. The mean (±SD) scores were 10.88 ± 6.02 for knowledge, 15.46 ± 3.15 for attitude, and 33.13 ± 5.19 for practice. In multivariable analyses, primary school education or below (*β* = −2.626, 95% CI: −4.593 to −0.659, *p* = 0.009), diagnosis of oesophageal, gastric cardia, or stomach cancer (*β* = −1.865, 95% CI: −3.278 to −0.452, *p* = 0.010), colorectal cancer (*β* = −1.670, 95% CI: −3.299 to −0.041, *p* = 0.045), and the use of nutritional supplements (*β* = 1.850, 95% CI: 0.813 to 2.888, *p* < 0.001) were associated with knowledge scores. Knowledge scores (*β* = 0.110, 95% CI: 0.066 to 0.154, *p* < 0.001), being a homemaker (*β* = −1.660, 95% CI: −2.834 to −0.486, *p* = 0.006), and the use of nutritional supplements (*β* = 0.558, 95% CI: 0.019 to 1.096, *p* = 0.042) were positively associated with attitude scores. For practice, only higher knowledge scores were independently associated with better nutritional practices (*β* = 0.359, 95% CI: 0.291 to 0.428, *p* < 0.001). Structural equation modeling showed that knowledge directly influenced attitudes (*β* = 0.194; *p* = 0.002) and practices (*β* = 0.546; *p* = 0.009).

**Conclusion:**

Patients diagnosed with malignant tumors exhibited inadequate knowledge and negative attitudes, although the practice was proactive, there was still room for improvement on specific practice. Targeted, multidisciplinary, and continuous nutritional education may be considered to support better nutritional awareness, patient-centered care, and overall quality of life.

## Introduction

Cancer, defined in medicine as a malignant tumor originating from epithelial tissue, is commonly referred to as any malignant tumor (1). As a major global public health concern, cancer has become the second leading cause of death worldwide, following cardiovascular diseases (2). From 2007 to 2017, the global incidence of cancer increased by 33%, with a rising number of new cases reported in nearly all countries each year. According to the International Agency for Research on Cancer (IARC), there were 19.29 million new cancer cases and 9.96 million cancer-related deaths globally in 2020, with projections estimating 28.4 million new cases by 2040. In China, the incidence and mortality rates of cancer are among the medium-high levels compared to 185 other countries or regions, making cancer the leading cause of death in the country (3, 4). Among oncology patients, the prevalence of malnutrition or risk of malnutrition ranges from 25 to 70%. Malnutrition can develop, persist, or worsen during the course of cancer and its treatment. However, malnutrition, particularly protein-energy deficits and muscle loss, often remains underdiagnosed and undertreated in oncology patients (5). Malnutrition significantly impacts mortality rates, with 10–20% of cancer patient deaths attributable to malnutrition rather than the malignancy itself (6).

The Knowledge-Attitude-Practice (KAP) theory is pivotal in shaping human health behaviors, as it posits that knowledge positively influences attitudes, which in turn shape individual practices (7, 8). Accordingly, the present study adopts the KAP theoretical framework to conceptualize and test the hypothesized pathways among nutritional knowledge, attitudes, and practices in patients with malignant tumors. Despite the critical role of nutritional management in cancer treatment, many cancer patients and their families often have inadequate knowledge, attitudes, and practices concerning nutrition. Some patients might lack fundamental understanding about the importance of nutrition or may not prioritize nutritional intake during treatment (9). Additionally, the discomfort and side effects associated with cancer treatment can lead to reduced appetite, further impacting nutritional intake (10).

Several recent studies have examined nutrition-related KAP in cancer or cancer-risk populations. For example, a study on patients with gastrointestinal cancer found substantial gaps in knowledge and weak translation of knowledge into dietary practice (9). Another KAP survey in gastric cancer patients reported that knowledge strongly influenced attitudes and practices, yet many participants still exhibited poor dietary behaviors (11). In caregivers of gastric cancer patients, moderate levels of nutrition knowledge and practice were observed despite generally positive attitudes, highlighting barriers in applying knowledge to supportive care (12). Meanwhile, reviews of the nutrition-cancer field emphasize persistent information gaps, such as limited awareness of nutrient composition, diet-treatment interactions, and standardized nutrition guidance in oncology care (13).

However, most existing studies have primarily focused on patients with specific cancer types such as gastrointestinal or gastric cancer, or on caregivers and survivors, rather than on a diverse malignant tumor population. Some recent investigations, including those by Zhi et al. (9) and Yu et al. (11), have applied structural equation modeling (SEM) to examine relationships among knowledge, attitudes, and practices in gastrointestinal cancers. Nevertheless, these studies were limited in scope, often constrained to single tumor sites and small, homogeneous samples. Few have explored the broader cross-cancer heterogeneity in nutritional KAP, or examined how sociodemographic and clinical characteristics jointly shape these associations. Moreover, differences in health literacy, cultural perceptions of nutrition, and health insurance coverage across cancer types and patient backgrounds remain underexplored, particularly in Chinese settings.

Therefore, this study aims to investigate the KAP regarding nutrition among malignant tumor patients in a Chinese hospital. Based on the KAP theory, it was hypothesized that (1) patients’ knowledge directly associated with both attitude and practice; (2) patients’ attitude directly associated with practice; (3) patients’ knowledge indirectly associated with practice. The findings aim to provide quantitative evidence to guide targeted educational interventions and improve nutritional management in oncology practice, thereby enhancing patients’ treatment outcomes and quality of life.

## Materials and methods

### Study design and participants

This cross-sectional study was conducted from October 16, 2023, to April 30, 2024, at author’s hospital, and included patients diagnosed with malignant tumors by a convenience sampling method. Participants were recruited from multiple inpatient departments across the hospital, primarily the Oncology Department, while patients hospitalized in other departments were also eligible to participate if they met the inclusion criteria. The study was approved by Ethics Committee of author’s hospital. All participants were informed about the study protocol and provided written informed consent to participate in the study. The inclusion criteria were: (1) patients with a confirmed pathological diagnosis of common malignant tumors; (2) aged between 18 and 80 years; (3) mentally clear and able to read and complete the questionnaire independently; (4) provided informed consent, acknowledging that their data would be used for scientific research. The exclusion criteria were: (1) patients with metabolic syndrome; (2) severe liver or kidney impairment; (3) severe internal diseases requiring special diets.

### Questionnaire

The questionnaire was designed and refined based on the recommendations of three experts, including one in geriatric medicine and two in palliative care. The questionnaire items were primarily developed based on the *Chinese Guidelines for Nutritional Therapy in Cancer Patients (2020)* issued by the Chinese Society of Nutritional Oncology, as well as the *Chinese Dietary Guidelines (2022)* published by the Chinese Nutrition Society. A pilot test involving 75 participants yielded a reliability coefficient (Cronbach’s alpha) of 0.912, and the internal consistency reliability coefficients for the knowledge, attitude, and practice subscales were 0.890, 0.801, and 0.843, respectively. The final questionnaire (Supplementary material), written in Chinese, comprised four dimensions of information collection. Basic information included gender, age, education, and type of work. The knowledge dimension consisted of 11 items, the attitude dimension included 10 items, and the practice dimension encompassed 8 items. For statistical analysis, scores were assigned based on the number of response options for each item. In the knowledge dimension, responses of understanding, partial understanding, and lack of understanding were scored 2, 1, and 0, respectively, with a total possible score ranging from 0 to 22. In the attitude dimension, responses of agree, partially agree, and disagree were scored 2, 1, and 0, respectively, with a total possible score ranging from 0 to 20. The practice dimension employed a five-point Likert scale, with scores ranging from 5 (always) to 1 (never), resulting in a total possible score ranging from 8 to 40. According to Bloom’s cut-off criteria, a score of 80% or higher of the maximum possible score was defined as indicative of good knowledge, a positive attitude, and proactive practice (14).

### Questionnaire distribution and quality control

The questionnaires were distributed to participants via a QR code from the Wenjuanxing platform. The questionnaires were distributed to eligible patients in inpatient wards during their hospital stay. If any problems were encountered during the response process, the doctors were responsible for giving a prompt explanation to the participants. After data collection was completed, the questionnaires were checked for quality by the members of the study team. Incomplete items, obvious logical errors, or a pattern of choosing exactly the same option to answer were considered invalid.

### Sample size

Sample size estimation was performed using Cochran’s formula: *n* = z^2^pq/e^2^ (with *z* = 1.96 for a 95% CI, p = expected proportion, *q* = 1 – p, and e = margin of error set at 0.05). Given the wide variation in reported prevalence across previous KAP studies in oncology settings, an expected proportion of 50% was adopted to yield the maximum required sample size. Based on this calculation, the estimated minimum sample size for the present study was 384 participants. This approach to sample size estimation is commonly applied in cross-sectional KAP studies (15, 16).

### Statistical analysis

Statistical analyses were conducted using SPSS 27.0 (IBM, Armonk, NY, United States) and AMOS 26.0 (IBM, Armonk, NY, USA). Continuous variables underwent a normality test, with the t-test for normally distributed data and the Wilcoxon Mann–Whitney test for non-normally distributed data when comparing two groups. For three or more groups with normally distributed continuous variables and uniform variance, ANOVA was used for comparisons, while the Kruskal–Wallis test was employed for non-normally distributed data. Univariate and multivariate linear regression analyses were performed to examine factors associated with knowledge, attitude, and practice scores, which were treated as continuous variables. This analytical approach was adopted to preserve the full variability of KAP scores and avoid potential information loss from score dichotomization. In the regression analysis, variables with a *p*-value < 0.05 in univariate analyses were entered into the multivariable models. The multicollinearity among factors that included in the regression analysis was assessed using variance inflation factors (VIF). The model fit for the linear regression analyses was evaluated using standard indicators, including *R*, *R*^2^, adjusted *R*^2^, and the standard error of the estimate. Structural equation modeling (SEM) was conducted to test the hypotheses of association among knowledge (K), attitude (A), and practice (P), and the KAP were used as latent variables, each measured by their corresponding questionnaire items. In addition, a post-hoc confirmatory factor analysis (CFA) was conducted to assess the construct validity of the questionnaire. The CFA was performed using data from all valid questionnaires included in the formal analysis. Model fit was evaluated using commonly accepted indices, including the chi-square to degrees of freedom ratio (CMIN/DF), root mean square error of approximation (RMSEA), incremental fit index (IFI), Tucker–Lewis index (TLI), and comparative fit index (CFI). A two-sided *p*-value less than 0.05 was considered statistically significant.

## Results

In this study, we initially collected 581 questionnaires. Data from four respondents who declined participation and 42 respondents who provided unclear responses to all knowledge items were excluded, resulting in 535 valid cases, achieving an effective response rate of 92.08%. Using the valid questionnaires, the CFA demonstrated acceptable construct validity (CMIN/DF = 3.036, RMSEA = 0.062, IFI = 0.836, TLI = 0.821, CFI = 0.835) (Supplementary Table S1). Among these respondents, 370 (69.16%) were male, with an average age of 60.67 ± 11.28 years. Additionally, 205 respondents (38.32%) had completed middle school education, 407 (76.07%) reported an average monthly household income of less than 5,000 yuan, 184 (34.39%) had been diagnosed with oesophageal, gastric cardia, or stomach cancer, and 115 (21.50%) had a relative with this type of malignancy. Regarding treatment, 170 (31.78%) and 339 (63.36%) respondents had undergone radiotherapy and chemotherapy, respectively, 246 (45.98%) had surgery, and 213 (39.81%) used nutritional supplements.

The mean knowledge, attitude, and practice scores were 10.88 ± 6.02, 15.46 ± 3.15, and 33.13 ± 5.19, respectively. Participants’ knowledge scores were more likely to vary depending on residence (*p* = 0.014), education (*p* < 0.001), type of work (*p* = 0.033), average monthly household income (*p* = 0.009), disease diagnosis (p < 0.001), presence of malignant tumor patients among relatives (*p* = 0.021), chemotherapy (*p* = 0.002), and nutritional supplements (*p* < 0.001). Meanwhile, their attitude scores were more likely to vary across average monthly household income (*p* = 0.018), presence of malignant tumor patients among relatives (*p* = 0.017), and nutritional supplements (*p* = 0.012). Furthermore, their practice scores were more likely to vary depending on residence (*p* = 0.007), education (*p* < 0.001), type of work (*p* = 0.001), average monthly household income (*p* = 0.034), type of health insurance (*p* = 0.005), and disease diagnosis (*p* = 0.014) (Table 1).

**Table 1:** Baseline characteristics of patients and KAP scores.

Characteristics	*N* (%)	Knowledge, Mean ± SD	*P*	Attitude, Mean ± SD	*P*	Practice, Mean ± SD	*P*
Total score	*N* = 535	10.88 ± 6.02		15.46 ± 3.15		33.13 ± 5.19	
Age	60.67 ± 11.28						
Gender			0.423		0.325		0.875
Male	370 (69.16%)	11.06 ± 6.24		15.57 ± 3.05		33.07 ± 5.27	
Female	165 (30.84%)	10.47 ± 5.47		15.21 ± 3.35		33.25 ± 5.01	
Residence			0.014		0.767		0.007
Urban	242 (45.23%)	11.53 ± 5.83		15.48 ± 2.99		33.81 ± 5.39	
Rural	293 (54.77%)	10.34 ± 6.12		15.45 ± 3.28		32.57 ± 4.86	
Education			<0.001		0.915		<0.001
Middle school	205 (38.32%)	10.96 ± 5.73		15.54 ± 3.17		33.24 ± 5.06	
High school/Technical secondary school	122 (22.80%)	11.78 ± 6.19		15.31 ± 3.26		34.38 ± 5.21	
College/University or above	74 (13.83%)	12.32 ± 5.72		15.68 ± 2.75		33.12 ± 5.03	
Type of work			0.033		0.282		0.001
Long-term stable employment (fixed job)	92 (17.20%)	12.47 ± 6.27		15.41 ± 2.94		34.61 ± 4.67	
Temporary work	22 (4.11%)	9.23 ± 5.56		14.55 ± 3.40		31.00 ± 4.85	
Homemaker	29 (5.42%)	10.52 ± 5.84		14.07 ± 2.84		33.83 ± 4.92	
Retired	141 (26.38%)	11.36 ± 5.55		15.48 ± 3.23		33.72 ± 4.75	
Unemployed	251 (46.92%)	10.21 ± 6.13		15.71 ± 3.15		32.36 ± 5.50	
Average monthly household income, yuan			0.009		0.018		0.034
<5,000	407 (76.07%)	10.43 ± 6.00		15.55 ± 3.22		32.84 ± 5.24	
5,000–10,000	104 (19.44%)	12.25 ± 5.75		14.90 ± 2.95		33.84 ± 4.92	
>10,000	24 (4.49%)	12.42 ± 6.59		16.29 ± 2.35		34.96 ± 5.00	
Type of health insurance			0.055		0.554		0.005
Basic medical insurance for urban employees	182 (34.02%)	11.52 ± 5.68		15.49 ± 3.02		34.07 ± 4.46	
Basic medical insurance for urban residents	130 (24.30%)	10.12 ± 5.82		15.70 ± 3.08		31.84 ± 5.35	
New rural cooperative medical insurance	209 (39.07%)	10.60 ± 6.20		15.31 ± 3.31		33.08 ± 5.37	
Other forms such as commercial insurance	14 (2.62%)	13.71 ± 8.08		15.14 ± 3.06		33.57 ± 5.72	
Disease diagnosis			<0.001		0.226		0.014
Lung cancer	139 (25.98%)	12.34 ± 5.83		15.50 ± 3.01		34.25 ± 4.88	
Oesophageal, gastric cardia, and stomach cancer	184 (34.39%)	9.74 ± 5.79		15.77 ± 3.04		32.77 ± 5.45	
Colorectal cancer	99 (18.50%)	10.08 ± 5.45		15.27 ± 3.23		32.30 ± 4.96	
Other	113 (21.12%)	11.62 ± 6.65		15.08 ± 3.39		33.04 ± 5.16	
Presence of malignant tumor patients among relatives			0.021		0.017		0.408
Yes	115 (21.50%)	12.06 ± 6.76		15.95 ± 3.38		33.52 ± 4.82	
No	376 (70.28%)	10.74 ± 5.83		15.38 ± 3.07		33.14 ± 5.24	
Not sure	44 (8.22%)	8.93 ± 4.89		14.91 ± 3.06		32.00 ± 5.66	
Underwent or currently undergoing radiotherapy			0.083		0.235		0.643
Yes	170 (31.78%)	11.65 ± 6.27		15.29 ± 3.02		32.96 ± 5.30	
No	365 (68.22%)	10.52 ± 5.87		15.54 ± 3.21		33.20 ± 5.14	
Underwent or currently undergoing chemotherapy			0.002		0.340		0.932
Yes	339 (63.36%)	11.45 ± 5.76		15.41 ± 3.08		33.17 ± 5.05	
No	196 (36.64%)	9.89 ± 6.32		15.55 ± 3.27		33.06 ± 5.44	
Underwent surgery			0.944		0.506		0.360
Yes	246 (45.98%)	10.83 ± 5.69		15.52 ± 3.19		32.89 ± 5.17	
No	289 (54.02%)	10.91 ± 6.29		15.41 ± 3.12		33.33 ± 5.21	
Not sure	0						
Ever taken or currently taking nutritional supplements			<0.001		0.012		0.112
Yes	213 (39.81%)	12.19 ± 5.83		15.93 ± 2.83		33.64 ± 4.82	
No	322 (60.19%)	10.01 ± 5.99		15.15 ± 3.31		32.79 ± 5.41	

The distribution of knowledge dimensions showed that the three questions with the highest number of participants choosing the “Uninformed” option were “Do you understand the Chinese Dietary Guidelines Food Pagoda?” (K11) with 61.50%, “Do you understand the specific ingredients, usage, and dosage of the nutritional supplements you are using?” (K10) with 55.14%, and “Do you understand that nutritional therapy needs to be integrated throughout the entire course of cancer treatment?” (K1) with 51.96% (Supplementary Table S2).

Regarding related attitudes, 47.85% disagreed that supplements can boost immunity and kill tumor cells (A3), 11.40% disagreed that drug or surgical treatments are more important than nutritional treatments (A1), and 6.54% disagreed with the need to have a food planner and implement it daily (A8) (Supplementary Table S3).

Responses to the practice dimension showed that only 40.19% always consume enough water (P4), only 40.56% always take the initiative to learn about diet and nutrition (P8), and only 43.55% always engage in appropriate exercise (P7) (Supplementary Table S4).

Univariate and multivariate linear regression analyses were conducted to identify factors associated with knowledge, attitude, and practice scores, which were treated as continuous variables. In the multivariate linear regression analysis for knowledge, lower knowledge scores were significantly associated with primary school education or below (*β* = −2.626, 95% CI: −4.593 to −0.659, *p* = 0.009), diagnosis of oesophageal, gastric cardia, or stomach cancer (*β* = −1.865, 95% CI: −3.278 to −0.452, *p* = 0.010), and colorectal cancer (*β* = −1.670, 95% CI: −3.299 to −0.041, *p* = 0.045). In contrast, the use of nutritional supplements was independently associated with higher knowledge scores (*β* = 1.850, 95% CI: 0.813 to 2.888, *p* < 0.001) (Table 2). For attitudes toward nutrition, multivariate linear regression analysis showed that higher knowledge scores were positively associated with attitude scores (*β* = 0.110, 95% CI: 0.066 to 0.154, *p* < 0.001). Being a homemaker was independently associated with lower attitude scores (*β* = −1.660, 95% CI: −2.834 to −0.486, *p* = 0.006), whereas the use of nutritional supplements was associated with more positive attitudes (*β* = 0.558, 95% CI: 0.019 to 1.096, *p* = 0.042) (Table 3). For practice, only higher knowledge scores were independently associated with better nutritional practices (*β* = 0.359, 95% CI: 0.291 to 0.428, *p* < 0.001), while other sociodemographic and clinical variables were not significantly associated with practice after adjustment (Table 4). There were no obvious multicollinearity (VIF > 5) among factors included in each multivariate linear regression model, and the Model fit was shown in Supplementary Table S5.

**Table 2:** Univariate and multivariate linear regression analysis of factors influencing knowledge dimension.

Characteristics	Univariate linear regression		Multivariate linear regression	
	Beta (95%CI)	*P*	Beta (95%CI)	*P*
Age	−0.039(−0.084–0.007)	0.094		
Gender				
Male	0.584(−0.522–1.690)	0.300		
Female	ref			
Residence				
Urban	1.191 (0.168–2.214)	0.023	−0.578(−1.911–0.754)	0.394
Rural	ref			
Education				
Primary school or below	−3.197(−4.884–−1.511)	<0.001	−2.626(−4.593–−0.659)	0.009
Middle school	−1.363(−2.943–0.216)	0.091	−1.224(−2.989–0.540)	0.173
High school/Technical secondary school	−0.546(−2.262–1.170)	0.532	−0.489(−2.246–1.267)	0.585
College/University or above	ref			
Type of work				
Long-term stable employment (fixed job)	2.260 (0.016–3.039)	0.044	1.042(−0.732–2.816)	0.249
Temporary work	0.201 (0.026–1.534)	0.122	−2.018(−4.590–0.554)	0.124
Homemaker	1.346 (0.543–3.332)	0.521	0.368(−1.860–2.596)	0.746
Retired	1.048 (0.623–1.762)	0.860	0.609(−0.877–2.095)	0.421
Unemployed	ref		ref	
Average monthly household income, yuan				
<5,000	−1.982(−4.448–0.484)	0.115		
5,000–10,000	−0.167(−2.825–2.492)	0.902		
>10,000	ref			
Type of Health Insurance*				
Basic medical insurance for urban employees	−2.198(−5.461–1.066)	0.186		
Basic medical insurance for urban residents	−3.599(−6.909–−0.289)	0.033		
New rural cooperative medical insurance	−3.111(−6.360–0.137)	0.060		
Other forms such as commercial insurance	ref			
Disease diagnosis				
Lung cancer	0.719(−0.757–2.194)	0.339	0.615(−0.890–2.120)	0.423
Oesophageal, gastric cardia, and stomach cancer	−1.875(−3.267–−0.483)	0.008	−1.865(−3.278–−0.452)	0.010
Colorectal cancer	−1.539(−3.142–0.065)	0.060	−1.670(−3.299–−0.041)	0.045
Other	ref		ref	
Underwent or currently undergoing radiotherapy				
Yes	1.129 (0.035–2.224)	0.015	0.201(−0.962–1.352)	0.732
No	ref			
Underwent or currently undergoing chemotherapy				
Yes	1.553 (0.499–2.606)	0.004	0.806(−0.324–1.934)	0.162
No	ref			
Underwent surgery				
Yes	−0.080(−1.106–0.946)	0.878		
No	ref			
Ever taken or currently taking nutritional supplements				
Yes	2.178 (1.150–3.207)	<0.001	1.850 (0.813–2.888)	<0.001
No	ref			

^*^Not included in the multivariate linear regression for limited sample size.

**Table 3:** Univariate and multivariate linear regression analysis of factors influencing attitude dimension.

Characteristics	Univariate linear regression		Multivariate linear regression	
	Beta (95%CI)	*P*	Beta (95%CI)	*P*
Knowledge	0.115 (0.071–0.158)	<0.001	0.110 (0.066–0.154)	<0.001
Age	0.013(−0.011–0.037)	0.275		
Gender				
Male	0.367(−0.212–0.946)	0.213		
Female	ref			
Residence				
Urban	0.028(−0.510–0.566)	0.918		
Rural	ref			
Education				
Primary school or below	−0.317(−1.215–0.580)	0.487		
Middle school	−0.139(−0.979–0.701)	0.745		
High school/Technical secondary school	−0.364(−1.277–0.549)	0.434		
College/University or above	ref			
Type of work				
Long-term stable employment (fixed job)	−0.296(−1.046–0.454)	0.438	−0.610(−1.348–0.127)	0.105
Temporary work	−1.164(−2.532–0.205)	0.095	−1.052(−2.384–0.280)	0.121
Homemaker	−1.640(−2.847–−0.433)	0.008	−1.660(−2.834–−0.486)	0.006
Retired	−0.234(−0.882–0.414)	0.478	−0.380(−1.012–0.253)	0.239
Unemployed	ref		ref	
Average monthly household income				
<5,000	−0.739(−2.034–0.556)	0.263		
5,000–10,000	−1.388(−2.784–0.008)	0.051		
>10,000	ref			
Type of health insurance				
Basic medical insurance for urban employees	0.346(−1.372–2.064)	0.692		
Basic medical insurance for urban residents	0.557(−1.185–2.299)	0.530		
New rural cooperative medical insurance	0.163(−1.547–1.873)	0.851		
Other forms such as commercial insurance	ref			
Disease diagnosis				
Lung cancer	0.417(−0.366–1.200)	0.296		
Oesophageal, gastric cardia, and stomach cancer	0.687(−0.052–1.425)	0.068		
Colorectal cancer	0.193(−0.658–1.044)	0.656		
Other	ref			
Underwent or currently undergoing radiotherapy				
Yes	−0.243(−0.814–0.332)	0.407		
No	ref			
Underwent or currently undergoing chemotherapy				
Yes	−0.144(−0.699–0.411)	0.611		
No	ref			
Underwent surgery				
Yes	0.104(−0.432–0.641)	0.702		
No	ref			
Ever taken or currently taking nutritional supplements				
Yes	0.781 (0.238–1.323)	0.005	0.558 (0.019–1.096)	0.042
No	ref			

**Table 4:** Univariate and multivariate linear regression analysis of factors influencing practice dimension.

Characteristics	Univariate linear regression		Multivariate linear regression	
	Beta (95%CI)	*P*	Beta (95%CI)	*P*
Knowledge	0.378 (0.312–0.444)	<0.001	0.359 (0.291–0.428)	<0.001
Attitude	0.168 (0.029–0.308)	0.018	0.025(−0.105–0.155)	0.191
Age	0.008(−0.031–0.048)	0.672		
Gender				
Male	−0.184(−1.140–0.771)	0.705		
Female	ref			
Residence				
Urban	1.239 (0.359–2.119)	0.006	0.154(−0.886–1.194)	0.771
Rural	ref		ref	
Education				
Primary school or below	−1.308(−2.767–0.151)	0.079		
Middle school	0.122(−1.244–1.488)	0.860		
High school/Technical secondary school	1.255(−0.229–2.740)	0.097		
College/University or above	ref			
Type of work				
Long-term stable employment (fixed job)	2.250 (1.025–3.475)	<0.001	1.336(−0.007–2.680)	0.051
Temporary work	−1.359(−3.593–0.876)	0.233	−0.996(−3.036–1.045)	0.338
Homemaker	1.469(−0.502–3.440)	0.144	1.380(−0.429–3.189)	0.135
Retired	1.358 (0.300–2.416)	0.012	0.851(−0.319–2.021)	0.154
Unemployed	Ref		ref	
Average monthly household income				
<5,000	−2.120(−4.254–0.013)	0.051		
5,000–10,000	−1.122(−3.422–1.178)	0.338		
>10,000	ref			
Type of health insurance				
Basic medical insurance for urban employees	0.500(−2.298–3.298)	0.726		
Basic medical insurance for urban residents	−1.733(−4.571–1.105)	0.231		
New rural cooperative medical insurance	−0.495(−3.280–2.290)	0.727		
Other forms such as commercial insurance	ref			
Disease diagnosis				
Lung cancer	1.208(−0.076–2.491)	0.065		
Oesophageal, gastric cardia, and stomach cancer	−0.273(−1.483–0.938)	0.659		
Colorectal cancer	−0.741(−2.136–0.653)	0.297		
Other	ref			
Underwent or currently undergoing radiotherapy				
Yes	−0.238(−1.186–0.709)	0.622		
No	ref			
Underwent or currently undergoing chemotherapy				
Yes	0.104(−0.812–1.020)	0.824		
No	Ref			
Underwent surgery				
Yes	−0.431(−1.316–0.454)	0.339		
No	ref			
Ever taken or currently taking nutritional supplements				
Yes	0.850(−0.049–1.748)	0.064		
No	ref			

Because there were no questionnaire modifications or additional structural path after CFA, the model fitness for SEM was equal to the CFA, indicated an acceptable model fit (CMIN/DF = 3.036, RMSEA = 0.062, IFI = 0.836, TLI = 0.821, CFI = 0.835). The results suggested that knowledge exerted a moderate direct effect on attitudes (*β* = 0.194, *p* = 0.002) and a stronger direct effect on practices (*β* = 0.546, *p* = 0.009) (Table 5 and Supplementary Figure S1), supporting the conceptual pathway that improved knowledge may positively influence both attitudes and health-related behaviors.

**Table 5:** Structural equation modeling (SEM) results.

Model paths	Standardized direct effects (95%CI)	*P*	Standardized indirect effects (95%CI)	*P*
Knowledge → Attitude	0.194 (0.126–0.299)	0.002		
Knowledge → Practice	0.546 (0.454–0.635)	0.009	0.002 (−0.031–0.022)	
Attitude → Practice	0.008 (−0.131–0.113)	0.865		

## Discussion

Patients diagnosed with malignant tumors exhibited inadequate knowledge and negative attitudes, although the practice was proactive, there was still room for improvement on specific practice. These findings provide important insights for clinical oncology practice, highlighting the urgent need to integrate structured nutritional education into cancer care pathways. Incorporating dietitians and clinical nutrition specialists as part of multidisciplinary oncology teams could help address these deficits and improve overall patient management.

Patients residing in urban areas and those with higher levels of education demonstrated better knowledge scores, likely due to increased access to health information and resources. Conversely, rural residents and those with lower education levels exhibited poorer knowledge, which may be attributed to limited access to nutritional education and health services. This finding aligns with previous studies that emphasize the disparities in health literacy between urban and rural populations (17, 18). Clinically, this suggests that nutritional counseling programs should be stratified, emphasizing outreach and simplified education for rural or low-literacy populations to reduce disparities and improve adherence to nutritional recommendations during treatment. The type of work also significantly affected knowledge and practice scores, with those in long-term stable employment showing better outcomes. This could be due to the stability and resources associated with steady employment, which facilitate better health management practices. Similarly, higher household income was associated with improved knowledge and practices, underscoring the impact of socioeconomic status on health behaviors (19, 20). From a clinical perspective, identifying patients at socioeconomic disadvantage allows clinicians to provide more intensive support, such as low-cost nutritional supplements or hospital-based dietary counseling, to mitigate financial barriers to treatment adherence.

Multivariate linear regression analyses further demonstrated that higher knowledge scores were significantly associated with better nutritional practices, suggesting that higher nutritional knowledge is associated with more favorable health-related behaviors. This association is also supported by the SEM results, which demonstrated a direct effect of knowledge on both attitudes and practices, whereas the direct effect of attitudes on practices was not statistically significant. Although the CFA and SEM demonstrated an acceptable overall model fit, several fit indices did not reach optimal thresholds. This may be partly explained by the heterogeneity of the study population, which included patients with different cancer types, treatment stages, and sociodemographic characteristics, as well as the use of self-reported questionnaire items reflecting real-world clinical settings. These findings are consistent with existing literature, indicating that better-informed patients are more likely to engage in health-promoting behaviors (21, 22).

Compared with previous studies, our findings both corroborate and extend existing evidence. For instance, Zhi et al. (9) and Yu et al. (11) reported that nutrition knowledge significantly influences attitudes and practices in patients with gastrointestinal cancer, which aligns with our SEM results demonstrating a direct positive pathway from knowledge to both attitudes and practices. However, the magnitude of these associations in our study was greater, possibly because our sample included a more heterogeneous population encompassing multiple cancer types. Unlike prior single-cancer studies, we also observed that socioeconomic and insurance-related variables were significant determinants of knowledge and practice scores, suggesting that structural and policy-level factors may mediate patients’ nutritional behaviors. Moreover, while Luo et al. (12) found that caregivers often demonstrate moderate knowledge but good attitudes, our patient sample showed uniformly suboptimal levels across all three KAP dimensions, indicating a more substantial educational gap among patients themselves. These differences underscore the need for individualized nutritional interventions tailored not only to cancer type but also to socioeconomic context and health literacy levels. This reinforces the clinical importance of knowledge-based interventions, educating patients is not merely informative but can lead to measurable behavioral improvements that impact treatment tolerance, recovery, and quality of life. Therefore, structured nutritional education should be embedded early in the cancer care continuum, ideally at diagnosis or during treatment initiation, to optimize outcomes.

Attitude scores were significantly associated with the presence of malignant tumor patients among relatives and the use of nutritional supplements. Patients with a family history of cancer and those using supplements had more positive attitudes toward nutrition. However, no significant differences in attitude scores were found based on residence, education, or work type, suggesting that while knowledge and practices may vary across these demographics, attitudes toward nutrition may be more uniformly influenced by personal and familial health experiences. The knowledge assessment revealed significant gaps, particularly in understanding the integration of nutritional therapy throughout cancer treatment, the specific ingredients and usage of nutritional supplements, and the Chinese Dietary Guidelines Food Pagoda. Over half of the respondents were uninformed about these critical aspects. Addressing these gaps is crucial, as inadequate knowledge can adversely impact patient outcomes. In clinical settings, hospitals should implement standardized, evidence-based nutritional education programs tailored to cancer patients. These could include bedside counseling by dietitians, integration of nutrition modules into discharge education, and follow-up through tele-nutrition platforms to ensure continuity of guidance (23, 24).

The attitude assessment revealed that while a majority of patients recognized the importance of nutrition, there were notable misconceptions. For instance, many believed that health supplements could boost immunity and kill tumor cells. Nearly half of the respondents disagreed with this statement, indicating a significant misunderstanding of the role of supplements. To address these issues, healthcare providers should correct these misconceptions through targeted educational campaigns emphasizing evidence-based information about supplements (25, 26). Oncologists, nurses, and pharmacists should jointly counsel patients on the safe and appropriate use of supplements, while integrating attitude-change strategies such as motivational interviewing and behavioral counseling into routine clinical follow-up (27, 28).

The practice assessment indicated variability in adherence to recommended nutritional practices among patients. For instance, less than half of the patients always ensured adequate daily water intake or engaged in appropriate physical activity. To bridge this gap, healthcare providers should establish robust support systems that facilitate adherence to nutritional guidelines (29, 30). These could include regular nutrition monitoring during treatment visits, digital reminders for hydration and diet tracking, and family-involved care plans to reinforce behavior change. Personalized nutrition plans should be dynamically adjusted according to treatment phase and patient tolerance to optimize clinical outcomes (31–34).

This study had several limitations. First, the cross-sectional design precludes establishing causality between knowledge, attitudes, and practices. Second, selection bias may have occurred due to the single-center design, inpatient recruitment, and the convenience sampling method, which may limit the representativeness of the sample and generalizability of the findings to other regions or populations. Third, the self-reported nature of the questionnaires may introduce information bias, including recall bias and social desirability bias, potentially leading to overestimation of knowledge or practices. Furthermore, while including patients with multiple cancer types in the present study improves representativeness, it also introduces heterogeneity that warrants cautious interpretation. To mitigate these biases, standardized questionnaires were used, data collection was conducted anonymously with clear instructions, and multivariable regression analyses were performed to adjust for relevant sociodemographic and clinical factors.

Based on the present findings, several clinical implications can be directly supported by the study data. First, the consistent association between higher nutritional knowledge and more favorable attitudes and practices suggests that routine nutritional education should be integrated into standard oncology care. Second, the observed knowledge gaps in areas such as nutritional therapy throughout cancer treatment, supplement use, and dietary guidelines highlight the need for targeted, structured nutrition counseling tailored to patients’ educational background and disease type. Third, the positive associations between nutritional supplement use and higher knowledge and attitude scores indicate that clinical encounters related to supplement use may provide valuable opportunities for nutrition-focused education. These recommendations are derived from observed associations rather than causal inferences and should be interpreted accordingly.

## Conclusion

In conclusion, patients diagnosed with malignant tumors exhibited inadequate knowledge and negative attitudes. Although the practice was proactive, there was still room for improvement on specific practice. From a clinical standpoint, these results emphasize that nutritional management should be an essential component of comprehensive cancer care. Early, individualized, and continuous nutritional education, delivered through multidisciplinary collaboration, may be considered as an important component of comprehensive cancer care to support better nutritional management and patient-centered outcomes.

## Data Availability

The original contributions presented in the study are included in the article/Supplementary material, further inquiries can be directed to the corresponding authors.

## Supplementary material

SUPPLEMENTARY FIGURE S1 SEM for KAP.
