# Supplementary figures and images for: Knowledge, attitudes, and practices regarding nutrition among patients with malignant tumors
Source: Front Nutr. 2026 Jan 21;13:1741346. doi: 10.3389/fnut.2026.1741346 (PMC12867899; doi:10.3389/fnut.2026.1741346)

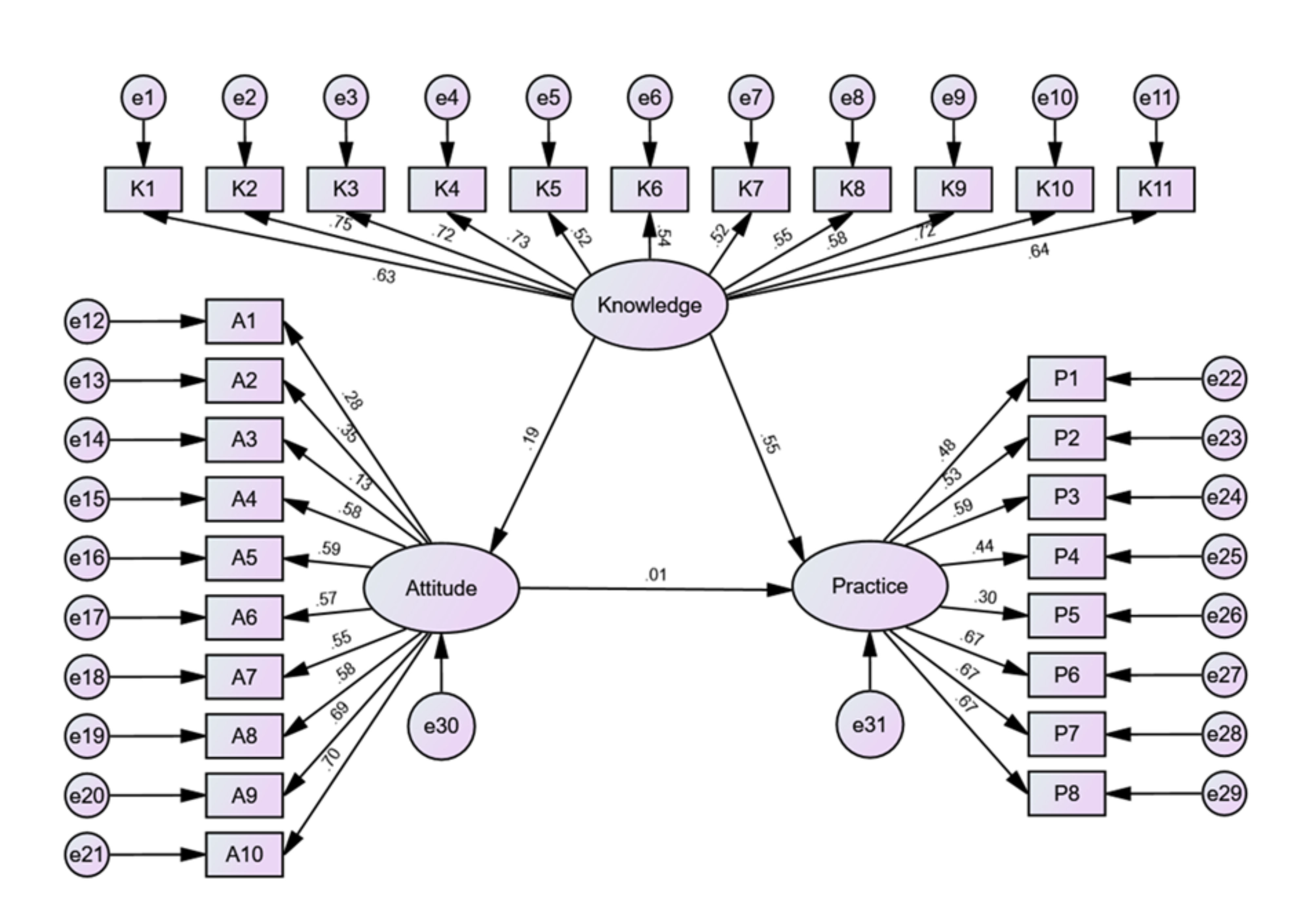

Supplement: SUPPLEMENTARY FIGURE S1 — SEM for KAP. [file Image_1.jpeg]
