# Supplementary material for: Knowledge, attitudes, and practices regarding nutrition among patients with malignant tumors
Source: Front Nutr. 2026 Jan 21;13:1741346. doi: 10.3389/fnut.2026.1741346 (PMC12867899; doi:10.3389/fnut.2026.1741346)
Supplement: Supplementary file 2 [file Table_1.doc]

Questionnaire Number：

| Dear Friend,  We are researchers from ** Hospital, and we sincerely invite you to participate in our research project. This study aims to understand the knowledge, attitudes, and practices of malignant tumor patients towards nutrition, in order to provide a basis for the development of scientific early intervention strategies, which may help more people in the future improve their health conditions. Your participation in this study is voluntary. If you agree to participate, please read the following instructions.  1. Please complete the questionnaire. There are no right or wrong answers, you just need to fill in according to the actual situation. If you have any questions during the answering process, you can ask us. Please submit it promptly after completion.  2. This study is a simple questionnaire survey, which will not cause harm to your physical and mental health, but it will involve some privacy issues, such as your gender, age, etc. We will strictly keep your information confidential and will not disclose it. Please feel free to fill it in.  3. As a participant, you can always learn about the information and research progress related to this study. If you decide to withdraw from the study, please let us know, and your data will not be included in the research results.  Finally, thank you sincerely for taking the time to support our scientific research amidst your busy schedule!  □ I have been informed and agree to use the collected data for scientific research.  Informed Consent Signature:  Date of Participation: Year Month Day | | | | | | | | | | | | | | |  |
| --- | --- | --- | --- | --- | --- | --- | --- | --- | --- | --- | --- | --- | --- | --- | --- |
| **Part 1 Basic Information** | | | | | | | | | | | | | | |  |
| 1. Your age: years old | | | | | | | | | | | | | | |  |
| 2. Your height: cm Your weight: kilograms | | | | | | | | | | | | | | |  |
| 3. Your gender: | a. Male  b. Female | | | | | | | | | | | | | |  |
| 4. Your residence: | a. Urban  b. Rural | | | | | | | | | | | | | |  |
| 5. Your education level: | a. Primary school and below  b. Middle school  c. High school/Technical secondary school  d. Junior college/Undergraduate  e. Master's degree and above | | | | | | | | | | | | | |  |
| 6. Your employment status: | a. Long-term stable employment (fixed job)  b. Temporary work  c. Homemaker  d. Retired  e. Unemployed | | | | | | | | | | | | | |  |
| 7. Your family's average monthly income: (RMB) | a. <5000  b. 5000-10000  c. 10001-20000  d. >20000 | | | | | | | | | | | | | |  |
| 8. Your medical insurance type: | a. Urban Employee Basic Medical Insurance  b. Urban Resident Basic Medical Insurance  c. New Rural Cooperative Medical Insurance  d. Commercial Insurance  e. Self-paid Medical Care | | | | | | | | | | | | | |  |
| 9. Your disease diagnosis: | a. Lung cancer  b. Esophageal, cardia, or gastric cancer  c. Colorectal cancer  d. Other | | | | | | | | | | | | | |  |
| 10. Do any of your relatives have the same type of malignant tumour (as selected in question 9)? | a. Yes  b. No  c. Unclear | | | | | | | | | | | | | |  |
| 11. Have you undergone or are you undergoing radiotherapy? | a. Yes  b. No  c. Unclear | | | | | | | | | | | | | |  |
| 12. Have you undergone or are you undergoing chemotherapy? | a. Yes  b. No  c. Unclear | | | | | | | | | | | | | |  |
| 13. Have you undergone surgery? | a. Yes  b. No  c. Unclear | | | | | | | | | | | | | |  |
| 14. Have you taken or are you taking nutritional supplements? | a. Yes  b. No  c. Unclear | | | | | | | | | | | | | |  |
| Simple Nutritional Assessment Questionnaire | | | | | | | | | | | | | | |  |
| 15. Have you experienced reduced appetite, digestive issues, chewing or swallowing difficulties leading to decreased food intake in the past three months? | | a. Severe decrease in food intake (0)  b. Marked decrease in food intake (1)  c. No change in food intake (2) | | | | | | | | | | | | |  |
| 16. Have you experienced weight loss in the past three months? | | a. Weight loss greater than 3kg (0)  b. Not sure (1)  c. Weight loss between 1 and 3kg (2)  d. No weight loss (3) | | | | | | | | | | | | |  |
| 17. Describe your level of physical activity. | | a. Bedridden or wheelchair-bound for a long time (0)  b. Can get out of bed or wheelchair but cannot go out (1)  c. Can go out (2) | | | | | | | | | | | | |  |
| 18. Have you experienced psychological trauma or acute illness in the past three months? | | a. Yes (0)  b. No (2) | | | | | | | | | | | | |  |
| 19. Describe any psychological issues you may have. | | a. Severe dementia or depression (0)  b. Mild dementia (1)  c. No psychological issues (2) | | | | | | | | | | | | |  |
| 20. What is your Body Mass Index (BMI) (kg/m2)? | | a. BMI less than 19 (0)  b. BMI between 19-21 (1)  c. BMI between 21-23 (2)  d. BMI greater than or equal to 23 (3) | | | | | | | | | | | | |  |
| **Part II Understanding of Nutritional Support for Cancer** | | | | | | | | | | | | | |  | |
| **1. Do you understand that nutritional therapy needs to be integrated throughout the entire course of cancer treatment?** | | | | | A.Well-Informed | | | B. Fairly Informed | | | | C. Uninformed | | | |
| **2. Do you understand which foods you need to eat for your specific disease?** | | | | | A.Well-Informed | | | B. Fairly Informed | | | | C. Uninformed | | | |
| **3. Do you understand which foods you need to avoid for your specific disease?** | | | | | A.Well-Informed | | | B. Fairly Informed | | | | C. Uninformed | | | |
| **4. Do you understand the main manifestations of malnutrition?** | | | | A.Well-Informed | | | B. Fairly Informed | | | | C. Uninformed | | | | |
| **5. Do you understand that malnutrition may worsen your condition?** | | | | A.Well-Informed | | | B. Fairly Informed | | | | C. Uninformed | | | | |
| **6. Do you understand that foods such as eggs and seafood can be consumed in moderation?** | | | | A.Well-Informed | | | B. Fairly Informed | | | | C. Uninformed | | | | |
| **7. Do you understand which foods are rich in protein, such as beans and bean products, dairy and dairy products, and red meat?** | | | |  | | A.Well-Informed | | | B. Fairly Informed | | | | C. Uninformed | | |
| **8. Do you understand which foods are rich in dietary fibre, such as vegetables, fruits, and whole grains?** | | | | A.Well-Informed | | | B. Fairly Informed | | | | C. Uninformed | | | | |
| **9. Do you understand that cancer patients should limit their intake of sugars (sweets, beverages, etc.)?** | | | | A.Well-Informed | | | B. Fairly Informed | | | | C. Uninformed | | | | |
| **10. Do you understand the specific ingredients, usage, and dosage of the nutritional supplements you are using?** | | | |  | | A.Well-Informed | | | B. Fairly Informed | | | | C. Uninformed | | |
| **11. Do you understand the Chinese Dietary Guidelines Food Pagoda?** | | | | A.Well-Informed | | | B. Fairly Informed | | | C. Uninformed | | | | | |
| **Part 3 Attitudes Towards Nutritional Support for Cancer** | | | | | | | | | | | | | | | |
| **1. I believe that medication or surgical treatment is more important than nutritional therapy.** | | | **A.Agree** | | | | **B.Partially Agree** | | | | **C.Disagree** | | | | |
| **2. I believe that certain foods, such as those traditionally considered "trigger foods," should be avoided.** | | | **A.Agree** | | | | **B.Partially Agree** | | | | **C.Disagree** | | | | |
| **3. I believe that health supplements can boost immunity and kill tumor cells.** | | | **A.Agree** | | | | **B.Partially Agree** | | | | **C.Disagree** | | | | |
| **4. I believe that malnutrition will affect my recovery.** | | | **A.Agree** | | | | **B.Partially Agree** | | | | **C.Disagree** | | | | |
| **5. I worry that an insufficient variety of food intake will affect my nutritional status.** | | | **A.Agree** | | | | **B.Partially Agree** | | | | **C.Disagree** | | | | |
| **6. I believe that emotions such as anxiety and fear will affect my appetite.** | | | **A.Agree** | | | | **B.Partially Agree** | | | | **C.Disagree** | | | | |
| **7. I believe that patients' meals should be prepared using methods such as steaming, stewing, or boiling.** | | | **A.Agree** | | | | **B.Partially Agree** | | | | **C.Disagree** | | | | |
| **8. I believe it is necessary to create a food plan listing daily foods and follow it accordingly.** | | | **A.Agree** | | | | **B.Partially Agree** | | | | **C.Disagree** | | | | |
| **9. I believe that hospitals need to promote knowledge about cancer nutrition more actively.** | | | **A.Agree** | | | | **B.Partially Agree** | | | | **C.Disagree** | | | | |
| **10. I believe that hospitals should provide dietary guidance to patients and their families.** | | | **A.Agree** | | | | **B.Partially Agree** | | | | **C.Disagree** | | | | |

| **Part 4 Behavioural Practices Regarding Nutritional Support for Cancer** | | | | | |
| --- | --- | --- | --- | --- | --- |
| **1. I will strictly adhere to the nutritional therapy as prescribed by my doctor.** | a. Always | b. Often | c. Sometimes | d. Occasionally | e. Never |
| **2. I will eat small, frequent meals and ensure a diverse intake of foods.** | a. Always | b. Often | c. Sometimes | d. Occasionally | e. Never |
| **3. I will be mindful of any adverse reactions that occur during nutritional therapy as an adjunct to cancer treatment.** | a. Always | b. Often | c. Sometimes | d. Occasionally | e. Never |
| **4. I will ensure adequate daily water intake (2000-3000ml).** | a. Always | b. Often | c. Sometimes | d. Occasionally | e. Never |
| **5. I will quit smoking and drinking alcohol after being diagnosed with cancer.** | a. Always | b. Often | c. Sometimes | d. Occasionally | e. Never |
| **6. I will regularly weigh myself and discuss my nutritional status with healthcare professionals.** | a. Always | b. Often | c. Sometimes | d. Occasionally | e. Never |
| **7. I will engage in appropriate physical activity.** | a. Always | b. Often | c. Sometimes | d. Occasionally | e. Never |
| **8. I will proactively learn about dietary and nutritional knowledge.** | a. Always | b. Often | c. Sometimes | d. Occasionally | e. Never |
